# Congenital Immature Teratoma of the Retroperitoneum

**Published:** 2013-07-01

**Authors:** Subhasis Roy Choudhury, Pinki R Debnath, Nitin Pant, Lalita Chowdhary

**Affiliations:** Department of Pediatric Surgery, Kalawati Saran Children's Hospital, Lady Hardinge Medical College, New Delhi- 110001

**Keywords:** Teratoma, Immature, Retroperitoneum

## Abstract

Congenital teratomas occur in extragonadal locations, the commonest site being the sacrococcygeal region. This report describes a rare case of antenatally detected, large, immature retroperitoneal teratoma. The diagnostic and therapeutic challenges of dealing with such a case have been discussed and the relevant literature reviewed. The recurrence of the tumor after gross surgical removal indicates a definitive role of administering chemotherapy in such a case.

## INTRODUCTION

Congenital teratomas tend to be extragonadal and histologically benign but carry a high mortality due to premature delivery of the baby or the development of hydrops fetalis [1]. Improvement in the results of treatment and survival rates of patients with teratomas diagnosed in utero raises important questions regarding the prognostic significance of immature germ cell elements which are frequently present in these tumors. This is because these immature elements often show maturation with time [2]. We report a case of congenital immature retroperitoneal teratoma that had been detected antenatally. The management of this rare condition is discussed and the relevant literature reviewed. 

## CASE REPORT

A full-term male neonate weighing 2.6 kg was referred for surgical treatment of an abdominal mass, which had been detected by an antenatal ultrasound scan (US) at 28 weeks of gestation. He was born by caesarian section, to a third gravida and second para mother. Abdominal examination revealed a firm central abdominal mass occupying almost all quadrants with restricted mobility of the mass. Plain X-ray abdomen showed the bowel gas shadows pushed to the left upper quadrant by a large space-occupying lesion. Postnatal US confirmed a large abdominal mass consisting of solid and cystic areas with foci of calcification. A computed tomographic (CT) scan of the abdomen at day five of life revealed a heterogeneous retroperitoneal mass (7cm× 5cm× 8.5cm) with calcification (Fig 1). The serum alpha-feto-protein (AFP) level was 484 ng/ml. A pan-systolic murmur was detected over the precordium. Doppler echocardiography showed a large muscular ventricular septal defect (VSD) with a left to right shunt and severe pulmonary hypertension. After consultation with the cardiologist, a decision was taken to proceed with the abdominal surgery. The child underwent laparotomy at 21 days of life. A large retroperitoneal tumor (8 cm×10cm) consisting of solid and cystic areas was found occupying mainly the right flank and central abdomen. The tumor was adherent to the surrounding structures namely the root of mesentery, superior mesenteric vessels, duodenum, colon, right kidney with renal vessels. However, there was no infiltration into any vessels or organs neither was any significant lymphadenopathy noted. Due to its friable nature, the tumor ruptured during removal resulting in intra-peritoneal spillage; however, gross complete excision of the tumor was performed (Fig. 2). Post-operatively the child developed congestive cardiac failure, which was treated with digitalis and diuretics. The child was discharged on the 10th postoperative day on full oral feeds. Histopathological sections from the tumor showed features of high-grade (grade III) immature teratoma with presence of immature neuroepithelial cells, glands, tubules, retinal tissue, hyaline cartilage and woven bone. Since the patient was a neonate and also had features of congestive cardiac failure, chemotherapy was not considered at that stage. The child underwent closure of the VSD at four months of age. At five months of age, the child presented with recurrence of the tumor, mainly on the left side of the abdomen, and a significantly elevated serum AFP level. The patient was re-operated for removal of the recurrent tumor. During surgery, a large recurrent tumor was found to be adherent to the retroperitoneal structures leading to incomplete removal and an iatrogenic duodenal injury. The child succumbed in the postoperative period to overwhelming sepsis.

**Figure:**
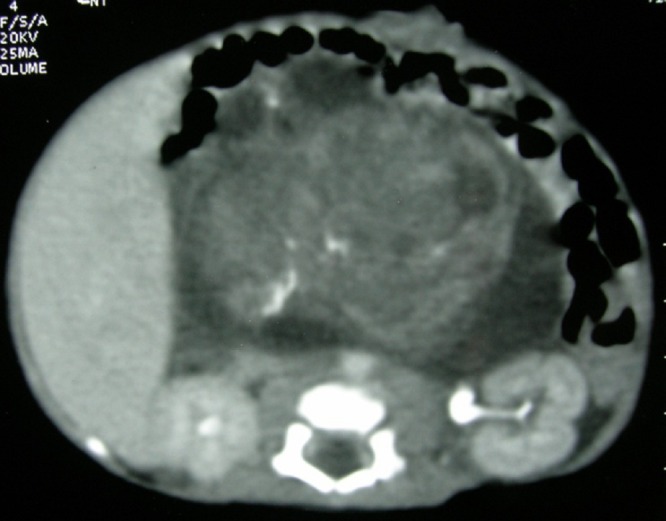
Figure 1: CT scan abdomen showing a large heterogeneous retroperitoneal mass with foci of calcification.

**Figure:**
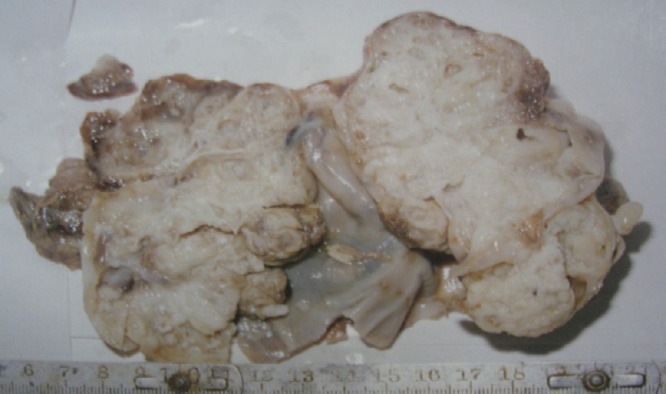
Figure 2: Cut surface of excised tumor.

## DISCUSSION

Teratomas are the commonest congenital neoplasms. The usual site for congenital teratomas is sacrococcygeal, the other sites being the mediastinum, head and neck, oropharynx, pericardium, and the retroperitoneum [3]. Lack et al [4] reported 3 cases of neonatal retroperitoneal teratoma; of these, two were immature and both succumbed to the disease. Antenatally detected immature teratoma of the retroperitoneum similar to our case has been extremely rarely reported [5, 6]. Generally, immature teratomas are associated with increased risk of tumor recurrence and malignant change. This does not appear to be the case with congenital teratomas. Histological tumor immaturity does not appear to correlate with malignancy in congenital teratoma [2]. Clinical stage and surgical resectibility are considered the most important prognostic factors [7]. The element of microscopic foci containing immature tissues varies from less than 10% in grade 1, 10 – 50 % in Grade 2, and more than 50% in grade 3 teratoma.

Immature elements like immature neuroepithelial cells, glands, tubules, retinal tissues were found histologically in the index case. The prognosis of neonatal teratomas is favorable with an 80-100% survival reported after surgical excision of the tumor and treatment of any recurrence [7, 8]. McKenney et al [2] in a series of 22 cases of congenital teratoma reported 100% survival and no recurrence after complete surgical resection with microscopic clearance, irrespective of histological tumor grade or immaturity; however, none of them was immature retroperitoneal teratoma. Tumor recurrence has been reported following spillage [8]. Some immature teratomas may continue to differentiate with time. Adjuvant chemotherapy was not considered in the index case being a neonate with associated congestive cardiac failure. However, for recurrence and for incomplete surgical excision, chemotherapy should be added in the treatment regimen (4-6 cycles of platinum based chemotherapy). Despite grossly complete surgical removal, the recurrence in our case could be attributed to microscopic seedling resulting from tumor rupture during surgery and not treating it with adjuvant chemotherapy. Serial serum AFP can be used as a maker to detect recurrence; Most of the recurrences occur within 7 months following excision [7], although recurrence can occur even up to 3 years after surgical excision. Therefore, a close follow-up of these patients is essential. 

In conclusion, the management of congenital immature retroperitoneal teratoma is complete surgical excision and microscopic clearance followed by close surveillance for any recurrence including serial serum AFP monitoring. For incomplete surgical excision or tumor spillage like in our case, post operative adjuvant chemotherapy should be given.

## Footnotes

**Source of Support:** Nil

**Conflict of Interest:** None

**Editorial Comment:** Neonatal tumors have a different biological activity and thus their prognosis is different from the children with tumors in post-infantile period. Though approximately 60% of the congenital tumors are benign, the incidence of malignancy is about 40%. Of all the congenital tumors, the extracranial teratomas have the highest frequency of occurrence of malignancy. The behavior of malignant teratomas/germ cell tumor in neonates and infants has been noted to be more aggressive with a higher incidence of recurrence and metastases in this age group. The same seems to be true for high-grade immature teratomasas reported in the index case. Taking these facts into consideration, the optimal therapy for high grade immature teratomas of congenital origin should not be just a single modality. A multimodality treatment approach would be more appropriate for long-term tumor-free survival without significant morbidity. No doubt that the tumor of these dimensions has to be surgically excised, but in case of intra-operative tumor spill, appropriate chemotherapy has to be administered postoperatively for improved outcomes. 

Intraoperative tumor spillage upgrades the stage of the tumor in almost all malignant tumors. Hence, much caution is warranted during dissection. When malignancy, especially a high grade, is confirmed, the patient with tumor warrants further treatment in the form of systemic therapy. The chemotherapy regimen most commonly used for immature teratomas is combination of Bleomycin, Etoposide and Cisplatin (BEP), which does not have any cardiotoxic effects. Platinum based chemotherapy has significantly improved the outcome of infants with malignant disease. Combination of Vinblastine, Actinomycin and Cyclophosphamide (VAC regimen) has also been frequently utilized for extratesticular immature teratomas of both grade 2 and 3 without any cardiotoxic effects. In neonates, the chemotherapy doses are as such administered with 50% reduction to decrease the toxic effects. Hence, the justification of not using chemotherapy in high-grade immature teratoma with intra-operative tumor spillage does not hold ground. 

Another major issue which needs to be addressed is recurrence. The high-grade (grade 3) pediatric immature teratomas, especially the extragonadal tumors have a much greater propensity for recurrence, as also incomplete resection. In this particular case, intra-operative tumor spillage is equivalent to microscopic residue of the disease. All recurrences of chemosensitive tumors must first be treated by systemic therapy as the extent of spread of disease can be assessed only macroscopically and the microscopic spread goes undetected, control of which can be done only by systemic therapy. Hence, upfront surgical intervention in the event of recurrence was inappropriate. 

The behavior of immature gonadal teratomas is different from immature extra-gonadal tumors. Contrary to the gonadal counterparts whose prognosis is not affected by the grade of the tumor and only surgical excisionis sufficient, the extragonadal immature teratomas require a judicious use of multimodality therapy depending on the grade of immaturity for long-term tumor free survival.

